# Cellular and molecular profiles of anterior nervous system regeneration in *Diopatra claparedii* Grube, 1878 (Annelida, Polychaeta)

**DOI:** 10.1016/j.heliyon.2021.e06307

**Published:** 2021-02-25

**Authors:** Mohd Ulul Ilmie Ahmad Nazri, Mohd Hafizi Mahmud, Basri Saidi, Mohd Noor Mat Isa, Zolkapli Ehsak, Othman Ross, Izwandy Idris, Wan Iryani Wan Ismail

**Affiliations:** a Cell Signalling and Biotechnology Research Group (CeSBTech), Faculty of Science and Marine Environment, Universiti Malaysia Terengganu, 21030, Kuala Nerus, Terengganu, Malaysia; b Biological Security and Sustainability Research Group (BIOSES), Faculty of Science and Marine Environment, Universiti Malaysia Terengganu, 21030, Kuala Nerus, Terengganu, Malaysia; c Centre of Medical Imaging, Faculty of Health Sciences, Universiti Teknologi MARA Selangor, Puncak Alam Campus, 42300, Bandar Puncak Alam, Selangor, Malaysia; d Malaysia Genome Institute, Jalan Bangi, 43000, Kajang, Selangor, Malaysia; e Imaging Centre, Faculty of Pharmacy, Universiti Teknologi MARA Selangor, Puncak Alam Campus, 42300, Bandar Puncak Alam, Selangor, Malaysia; f Faculty of Science and Marine Environment, Universiti Malaysia Terengganu, 21030, Kuala Nerus, Terengganu, Malaysia; g South China Sea Repository & Reference Centre, Institute of Oceanography & Environment, Universiti Malaysia Terengganu, 21030, Kuala Nerus, Terengganu, Malaysia

**Keywords:** Annelida, Nervous system, Early regeneration, Polychaete, Ultrastructure, Transcriptome, Neurodegenerative diseases, Micro CT scan, Ultramicrotome, Bioinformatics

## Abstract

The polychaete *Diopatra claparedii* Grube, 1878 is among those organisms successfully carrying out full body regeneration, including the whole nervous system. Thus, *D. claparedii* potentially can be regarded for the nervous system regeneration (NSR) study. However, data on the property of its nervous system and the NSR profile are still lacking. In this study, we investigated the morphology of *D. claparedii* anterior nervous system (ANS) and examined the cellular and molecular profiles on its early anterior NSR. The nervous system of *D. claparedii* consists of a symmetry brain with nerves branching off, circumpharyngeal connectives that connect the brain and nerve cord as well as obvious segmental ganglia. Moreover, we identified changes in the cellular condition of the ganglionic cells in the regenerating tissue, such as the accumulation of lysosomes and lipofuscins, elongated mitochondria and multiple nucleoli. Furthermore, mRNA of tissues at two regenerating stages, as well as intact tissue (non-regenerating), were sequenced with Illumina sequencer. We identified from these tissues 37,248 sequences, 18 differential expressed proteins of which upregulated were involved in NSR with noelin-like isoform X2 turned up to be the highest being expressed. Our results highlight the cellular and molecular changes during early phase of NSR, thus providing essential insights on regeneration within Annelida and understanding the neurodegenerative diseases.

## 1 Introduction

Nervous system regeneration (NSR), or also known as neuroregeneration, is defined as restoration and regrowth of the lost neuron or nerve tissue to regain normal function. In invertebrates, such as echinoderms, the regeneration capacity is remarkable as they can regenerate both internal and external parts, including the nervous system (Ortega and Olivares-Bañuelos, 2020). Neurogenesis is a process by which a new neuron is produced from a stem/precursor cell during the developmental process from juvenile to adult. However, in some adult insects, it was found that persistent neurogenesis occurs in the mushroom bodies which are known to function in learning and memory (Cayre et al., 2002). Furthermore, a study conducted on an adult serpulid polychaete has demonstrated the outgrowth of a cerebral ganglion as the first feature established during neurogenesis (Brinkmann and Wanninger, 2009). On the other hand, the second process, neuroplasticity, is an adaptation of the brain towards environment enhancement in terms of structure and function (Enciu et al., 2011). In invertebrates, especially mollusks, and insects, neuroplasticity has been reported to occur for axon sprouting and synapse formation (Pyza, 2013). Thus, the cause of neurodegeneration, on the contrary, might be due to the absence of the neuroplasticity process.

The capacity for neuroregeneration varies widely among taxa. Invertebrates are known to regenerate the entire body while phylogenetically primitive vertebrates such as lizards and zebrafish selectively regenerate certain parts. Lizards such as geckos and skinks, for example, regenerate the losing tail once the tail drops. However, the regenerated tail remains different because the original spinal cord is replaced by an epithelial tube (Alibardi, 2009). Meanwhile, the zebrafish, *Danio rerio* is widely used as an animal model to study neurodegenerative disease. The zebrafish is able to repair lesions in the brain, regenerate tissue, and neurons in the spinal cord, brain, and retina, but only if the residential glial cells remain (Gemberling et al., 2013; Nazri et al., 2019). However, the regeneration does not occur *de novo*. Thus, several organisms, mostly invertebrates, maintain the ability to regenerate certain parts for survivability. Moreover, some invertebrate species are capable of regenerating entirely to become new individuals, for example, flatworm (Platyhelminthes) and marine worms from class Polychaeta (phylum Annelida). In polychaetes, NSR begins with the outgrowing of nerve fibre from the nerve cord of the original body that later formed the brain and other nerves (Weidhase et al., 2015).

Polychaetes represent almost 65% of all annelid species (Anderson, 2013) making them an incredibly diverse and abundant marine invertebrates group. The class was erected by Grube (1850) and, at present, consisting of 85 families. Most of them are benthic, and some are pelagic, which can be found in marine, brackish water; a small number of species live in freshwater and even less, terrestrial (Thorp and Rogers, 2014). Polychaetes are separated into two large orders, Errantia and Sedentaria based on their morphology and behaviour. Errant polychaetes are highly mobile and free-living while sedentary polychaetes are typically tubiculous. Their adult size varies from less than 1 mm up to 6 m. Their colours are often vibrant and iridescent. They feed on smaller prey or by trapping food particles by ciliary action. Polychaetes reproduce sexually with separate sexes and some asexually with a high number of variabilities. Fertilisation mainly occurs externally in the water and results in either free-swimming larvae or direct development (Rouse and Pleijel, 2001). In Malaysia, a recent literature study in 2013 found a total of 64 species from 31 families have been identified in Malaysia from different environments (Idris and Arshad, 2013). The number of species in Malaysia is believed to increase since then.

The polychaete's nervous system consists of three major components, i.e. brain, nerve cord, and peripheral nerve, which are further divided into central (CNS) and peripheral nervous system (PNS). CNS in polychaetes consists of the brain and nerve cord which is supported by segmental ganglia. Neurons or perikarya of the brain surround the structure with a compact central mass of neuropil located in the centre. Some nerves innervate upwards to palp (called palp nerves) and downward towards the nerve cord (called circumpharyngeal connectives) of the CNS. The first nerve cord that connects with circumpharyngeal connectives is called the subpharyngeal ganglion. The nerve cord is extended from this ganglion towards pygidium in either double- or triple-stranded arrangement. Each body segment consists of a pair of ganglia that are connected by commissures, while the ganglion is connected to other ganglions from the next body segment by connectives. In PNS, lateral nerves branch away from ganglion from each segment towards the lateral bodyside (Rouse and Fauchald, 1997).

*Diopatra claparedii* (Polychaeta: Onuphidae) is capable of regenerating anterior and posterior parts (Rouse and Pleijel, 2001). The regeneration process begins with wound healing, followed by dedifferentiation of cells near the wounded site to become blastema cells (undifferentiated cells that behave like stem cells). After that, blastema cells will redifferentiate into specialised cells to initiate segmentation until it reaches into a complete regeneration (Bely, 2014). The early phase of regeneration involves wound healing, blastema formation, and the early stage of segmentation. We speculate that the transition between blastema formation to nerve tissue development is crucial and less reported. The process of regeneration (neurogenesis), at this stage, requires the presence of a nervous system that stimulates cell proliferation in blastema (Reuter et al., 1996). Furthermore, a new nervous system arises from the nervous system from the original body before any other structures appear (Yoshida-Noro et al., 2000). Hence, there are many reports on molecular studies in regeneration in polychaetes. However, studies on NSR are still lacking. To elucidate potential molecular factors involved in NSR, we conducted RNA-sequencing (RNA-seq) in two regeneration stages, including before (intact polychaete) and after regenerative took place. This approach was selected to obtain transcriptome information and to determine differentially expressed proteins at the early regeneration stage of the anterior region. Therefore, our investigation findings thus give additional information on regeneration aspects of annelids focusing on the nervous system regeneration, which may be beneficial in understanding the neurodegenerative diseases.

## 2 Materials & methods

### 2.1 Animals

**Figure 1:**
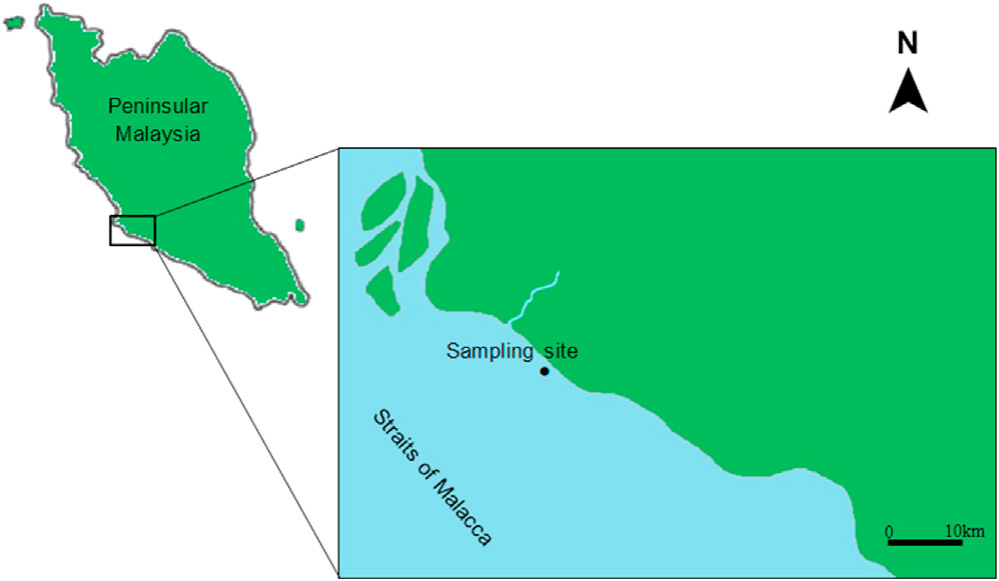
Map of the study with the location of sampling site in Morib Beach, Banting, western Peninsular Malaysia.

The polychaetes were collected in the intertidal mudflat area in Morib Beach, Banting (Figure 1), west coast of Peninsular Malaysia (2.7495̊ N, 101.4426̊ E). The worms were removed by digging the sandy mud sediment to obtain the longest possible tubes. Each collected tube was checked to make sure the worm is present. The worms are susceptible to sediment vibration caused by human footprint and will burrow deeper into the sediment. Hence, the minimum length of *D. claparedii* that can be used for the experiment is 5 cm when relaxing. For the laboratory acclimatisation, all collected *D. claparedii* were maintained in the aquaria containing sediment from the sampling site for two weeks, at 28 °C and 23–25 ppt salinity of artificial seawater (Aquaforest; Brzesko, Poland). The worms were fed with commercial sinking fish pallet *ad libitum.* During the habituation period, the polychaetes were allowed to regenerate the posterior section themselves since they were accidentally amputated during collection. In each experimental procedure, the polychaetes were immobilised with 4% MgCl2.6H2O.

### 2.2 Cresyl violet staining

Intact polychaetes were fixed in 10% formalin mixed with artificial seawater overnight (Mackie, 1994). Then, the fixed specimens were dehydrated in ascending alcohol (70–100%) to remove all traces of water, followed by clearing in xylene. The anterior section (prostomium and peristomium) and all external appendages were then removed. The anterior part was then embedded in paraffin wax prior to sectioning. The paraffin block was sectioned to 5 μm of thickness using a microtome and then proceed to the standard histological slide preparation before staining. The slides were then immersed in 0.5% cresyl violet, which stained the Nissl bodies in the neurons for 2 min and washed again with distilled water followed by dehydration by 70% ethanol and twice absolute ethanol for 2 min, respectively and finally with a 5 min wash in xylene. The sections were covered up with a coverslip and viewed under a light compound microscope.

### 2.3 Immunohistochemistry combining with confocal laser scanning microscopy

The anterior part of intact polychaete was fixed in 4% paraformaldehyde for 12 h, before overnight incubation in 15% sucrose solution (to sustain the tissue shape). The specimen was then mounted on OCT medium before sectioning by cryostat Leica CM1950 (Leica Biosystems, Wetzlar, Germany). The specimen was sectioned 8 μm thickness horizontally in dorsal position for the brain and ventral position for ventral nerve cord. The sections then were transferred onto a superfrost glass slide (Thermo Scientific, Waltham, MA, USA). The staining procedure was conducted according to the protocol from Cell Signaling Technology with several modifications. The sections were fixed in an ice-cold (-80 °C) methanol for 10 min, followed by two washes with PBS for 5 min in each wash. Next, the sections were incubated in 3% H_2_O_2_ diluted in methanol for 10 min at room temperature (RT) before two washes by PBS. The samples were then blocked for 1 h at RT by PBST-NGS (0.1 M PBS, goat serum (Sigma), triton-X with the ratio of 200:4:1). The samples were then incubated with primary antibody (monoclonal anti-acetylated α-tubulin by Santa Cruz, Dallas, TX, USA; dilution 1:250 in PBST-NGS) in a humid dark box at 4 °C. The sections were then washed twice with PBS for 5 min each and incubated with secondary antibody (polyclonal mouse IgG conjugated DyLight488 by GeneTex, Irvine, CA, USA; dilution 1:500) for 30 min at RT in the same dark box. Finally, the samples were washed before being mounted with an antifading mounting medium and covered with a coverslip. The slides containing sections were viewed with a Leica TCS SPE confocal microscope (Leica Biosystems) at 488 nm wavelength.

### 2.4 Micro-computed tomography scanning (Micro-CT)

Intact polychaetes were dehydrated in a series of ascending ethanol for 45 min each (90% twice, 96% twice) before they were dried with hexamethyldisilazane (HMDS) for 2 h. The specimens were left overnight prior to tomography scanning. Image of the specimen was acquired by using SkyScan 1176 microtomography (Bruker micro-CT; Kontich, Belgium) in medical imaging laboratory at Faculty of Health Sciences, UiTM Puncak Alam, Selangor. Specimens were scanned at 9 μm resolution. A 50 kV voltage with a current of 250 μA was applied, and scans were conducted for a full rotation of 360̊. Raw images (.tif) acquired from the scanning were reconstructed into cross-sections (.bmp) by using NRecon (SkyScan) software from the total number of 360̊ projection images. To check the details of the scanned specimen (externally and internally), we viewed the reconstructed image using DataViewer before we proceeded with 3D volume rendering.

### 2.5 Regeneration experiment

In the beginning, polychaetes were amputated at either segment four or 10 to observe the highest successful regeneration (Pires et al., 2012). We concurred that regeneration was more successful from those amputated at segment four based on preliminary study and thus proceeded with this segment for further experiments. Prior to amputation, polychaetes were removed from their tubes by a blunt end stick from the posterior opening and anaesthetised at 4% MgCl2.6H2O. Amputation was made by using a scalpel under a stereomicroscope. The posterior ends were pushed back inside the tube by forceps with the anterior part entered first. The tube was then located in a cylinder plastic container (7 cm diameter x 15 cm height) with sediments inside. The container was fully submerged in an aquarium that contained artificial aerated seawater. At this stage, they were left without food since they had no mouth. Images of successfully regenerated polychaetes at certain days after amputation until day 60 were captured.

### 2.6 Ultrastructure investigation

This experiment was conducted following the method provided by Imaging Center, Faculty of Pharmacy, UiTM Puncak Alam, Selangor with slight modifications.Blastema tissue appeared on day 6 p.a (D6 polychaete) was involved in this experiment. Besides blastema tissue, the investigation was also conducted on the ventral nerve cord. It was speculated that ganglion in the ventral nerve cord near the amputation site might play a crucial role in triggering NSR. Hence, two anterior-most segments from D6 polychaete, one anterior-most segment from the original body connecting with a new segment of D14 polychaete, and segments 4 to 5 from INT polychaete were amputated and fixed in 4% glutaraldehyde overnight at 4 °C. The samples were then washed three times in 0.1 M cacodylated buffer for 10 min each. Next, the samples were fixed with 1% osmium tetroxide for 2 h at 4 °C. The samples were then washed with cacodylate buffer three times for 10 min each at RT. Later, the samples were dehydrated in the series of ascending concentrations of acetone (30, 50, 75, 95% for 10 min each) and lastly 100% three times for 15 min each. The samples were then infiltrated with a series of resin: acetone mixture [resin: acetone (1 : 1); resin: acetone (3 : 1)] for 1 and 2 h respectively, followed by an overnight in resin only]. Next, the resin was aspirated out, and the newly prepared resin was introduced for 2 h. The specimens were then transferred into beam capsules (later called specimen block) and filled up with resin and polymerised in an oven at 60 °C for 48 h. Next, the specimen blocks were trimmed and sectioned using an ultramicrotome (Leica EM UC7; Wetzlar, Germany). The sections were transferred onto a copper grid, stained and counterstained with 2% uranyl acetate and lead citrate for 10 min, respectively. The grids finally were examined under a transmission electron microscope (Tecnai G2 20; Oregon, USA).

### 2.7 Total RNA isolation

Tissues involved in RNA isolation were explained in ultrastructure experiment with n = 2 for each group. The isolation procedure was conducted according to the protocol by the Nucleospin® RNA Plus (Macherey-Nagel, Düren, Germany). The first step involved the crushing of the tissue in liquid nitrogen using mortar and pestle. The crushed tissues were carefully inserted inside frozen-RNAse-free 1.5 ml tubes and lysed with 350 μL lysis buffer. Next, the homogenised lysate was transferred to another tube in a column provided in the kit and centrifuged for 30 s at 11,000 rpm. Then, the column was discarded, and the flow-through was mixed with 100 μL binding solution by moderate vortexing. The whole lysate was transferred to another column pre-assembled with a collection tube and centrifuged for 15 s at 11,000 rpm. After that, 200 μL washing buffer #1 (provided) was added to the column and centrifuged for 15 s at 11,000 × *g.* The flow-through was discarded, and the column was placed in a new collection tube. Next, 600 μL of washing buffer #2 (provided) was added into the column and was centrifuged at 11,000 × *g* for 15 s before the step was repeated for another 250 μL washing buffer #2 that was centrifuged for 2 min at 11,000 rpm. The final step was the elution of RNA where 30 μL of RNAse-free water was added inside the previous tube and centrifuged at 11,000 × *g* for 1 min before the step was repeated once more. The quality of RNA was assessed on a 2100 Bioanalyzer (Agilent Inc, Santa Clara, CA, USA) and agarose gel electrophoresis. The yields were stored in -80 °C until use.

### 2.8 Library preparation and RNA-seq

All steps for preparing libraries and sequencing were conducted at the Malaysia Genome Insitute, Malaysia by referring to TruSeq Stranded mRNA Sample Protocol (Illumina, San Diego, CA, USA). The poly-A containing mRNA from total RNA that was previously extracted was purified by using poly-T oligo attached with magnetic beads. After that, the mRNA was fragmented into small pieces by divalent cations under elevated temperature. These fragments then were copied into the first cDNA using reverse transcriptase and random primers. Actinomycin D was added to prevent the non-genuine DNA-dependent synthesis, and at the same time allowing RNA-dependent synthesis to improve strand specificity. The strand specificity was achieved by replacing dTTP with dUTP in the second strand marking mix and followed by second-strand cDNA synthesis using DNA polymerase 1 and RNAse H. The inclusion of dUTP in the second-strand synthesis reduced the second strand during amplification. The blunt fragments tend to ligate to each other during the adapter-ligation reaction; thus, a single ‘A’ nucleotide was added to the 3′ end of the fragments. The next step involved the enrichment of the DNA fragments by polymerase by PCR and was purified to create the final cDNA library. Finally, the cDNA libraries for each group were sequenced on an Illumina HiSeq 2000 (Illumina, San Diego, CA, USA) and data obtained were generated in FASTQ format.

### 2.9 Bioinformatic analysis

To avoid the generation of spurious contigs by phix-174 contamination, RNA-seq reads (FASTQ) which have forward and reverse reads for each sample, were trimmed and mapped using Bowtie 2 (Langmead and Salzberg, 2012), also known as cleaned reads. In *de novo* assembly, the cleaned reads were assembled using Trinity (GitHub, San Francisco, CA, USA), and genes with differential expression were identified using Empirical Analysis of Digital Gene Expression in R software (edgeR) (Bioconductor). A p-value < 0.05 was considered significantly different between groups. Next, the transcripts were filtered by CDHit to eliminate the redundant transcripts, and the non-redundant transcripts were blasted for protein profiles (.xml). The blasted transcripts were then loaded in Blast2GO software for mapping to retrieve GO terms, and annotation to select reliable biological processes of each protein.

### 2.10 Statistical analysis

Statistical analysis was only involved in the analysis of sequencing data. The differential expression of transcripts from each group was determined by pairwise comparisons using edgeR software from Bioconductor. The significant level was decided at *p*-value < 0.05.

## 3 Results

### 3.1 Characterisation of the anterior nervous system

**Figure 2:**
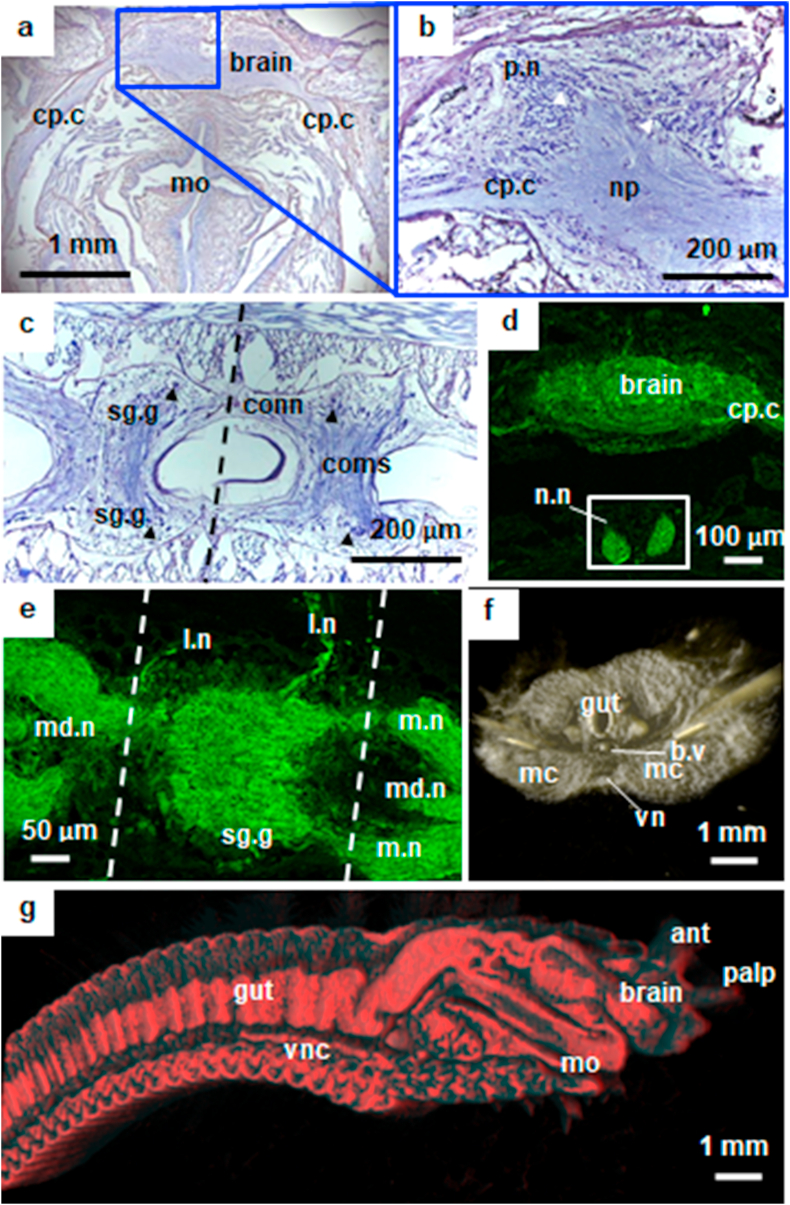
Anterior nervous system morphology of *D. claparedii.* a-c, Cresyl violet stained sections. d-e, anti-acetylated α-tubulin stained sections. f-g, false-colour 3D images captured by micro-CT. (a) Dorsal view of horizontal section at prostomium. Brain has a symmetrical shape with left and right circumpharyngeal connectives (cp.c) connecting towards posterior. (b) Magnified left side of the brain. Two nerves were spotted, i.e. palp (p.n) and cp.c. Neuropil (np) consists of nerve fibre and from the surrounding perikarya (white arrowhead). (c) Section from ventral side, horizontally. The dotted line separating two equal segments where each segment consisted of a pair of segmental ganglia (sg.g), the nerve connecting the two ganglia, called commissure (coms). Connective (conn) is a nerve connecting two ganglia from different segment. Note as well the perikarya (black arrowhead) forming a ganglion. (d) Antibody stained the brain and additional nuchal organs (box). nuchal nerves (n.n) were also seen. (e) New structures were spotted from the immunoreaction, i.e. lateral nerve (l.n) and median nerve (md.n). (f) Virtual axial cut of segment six from anterior. The nerve cord was located ventrally (vnc) below gut and ventral blood vessel (b.v). (g) Lateral view of sagittal plane in the middle of polychaete's body. The brain and vnc were separated far, but connected by cp.g (not shown). vnc can be seen run along the body towards posterior just above the skin. ant - antenna, m.n - main nerve, mc - muscle, mo - mouth, S1–S4 - segments.

For the first time, the morphology of the ANS in *D. claparedii* is fully described. Our findings from anterior regions stained by cresyl violet and labelled by anti-acetylated α-tubulin exposed the brain, branches of nerve and nerve cord. The brain is symmetrically divided into two attached hemispheres; a pair of palp nerves branching anteriorly and a pair of circumpharyngeal nerves branching posteriorly (Figure 2a). Neuron cell bodies known as perikarya (sing., perikaryon) were accumulated around the brain with nerve fibre mass in the middle, known as neuropil (Figure 2b). The double-stranded nerve cord was observed along the ventral side with a pair of segmental ganglia connected by commissure. In addition, ganglion from each segment was connected by a connective (Figure 2c). All these structures form the CNS of *D. claparedii*.

Our finding from anti-acetylated α-tubulin labelling confirmed the nerve structures as mentioned above with additional nuchal organs, nuchal nerves and lateral nerves of PNS (Figure 2d). Interestingly, the nerve observed under confocal laser scanning microscope also revealed an additional median nerve in the nerve cord, suggesting the possibility of *Diopatra claparedii* has a triple-stranded nerve cord (Figure 2e). Furthermore, we conducted micro-computed tomography scanning (micro-CT) to confirm the location of these nerve structures in *D. claparedii*. *D. claparedii* brain is located at the dorsal region of the head known as the prostomium, just above the pharynx. Meanwhile, the nerve cord is located ventrally started at around 7^th^ segment (Figures 2f, 2g).

### 3.2 Cellular evidence of early anterior NSR in *D. claparedii*

**Figure 3:**
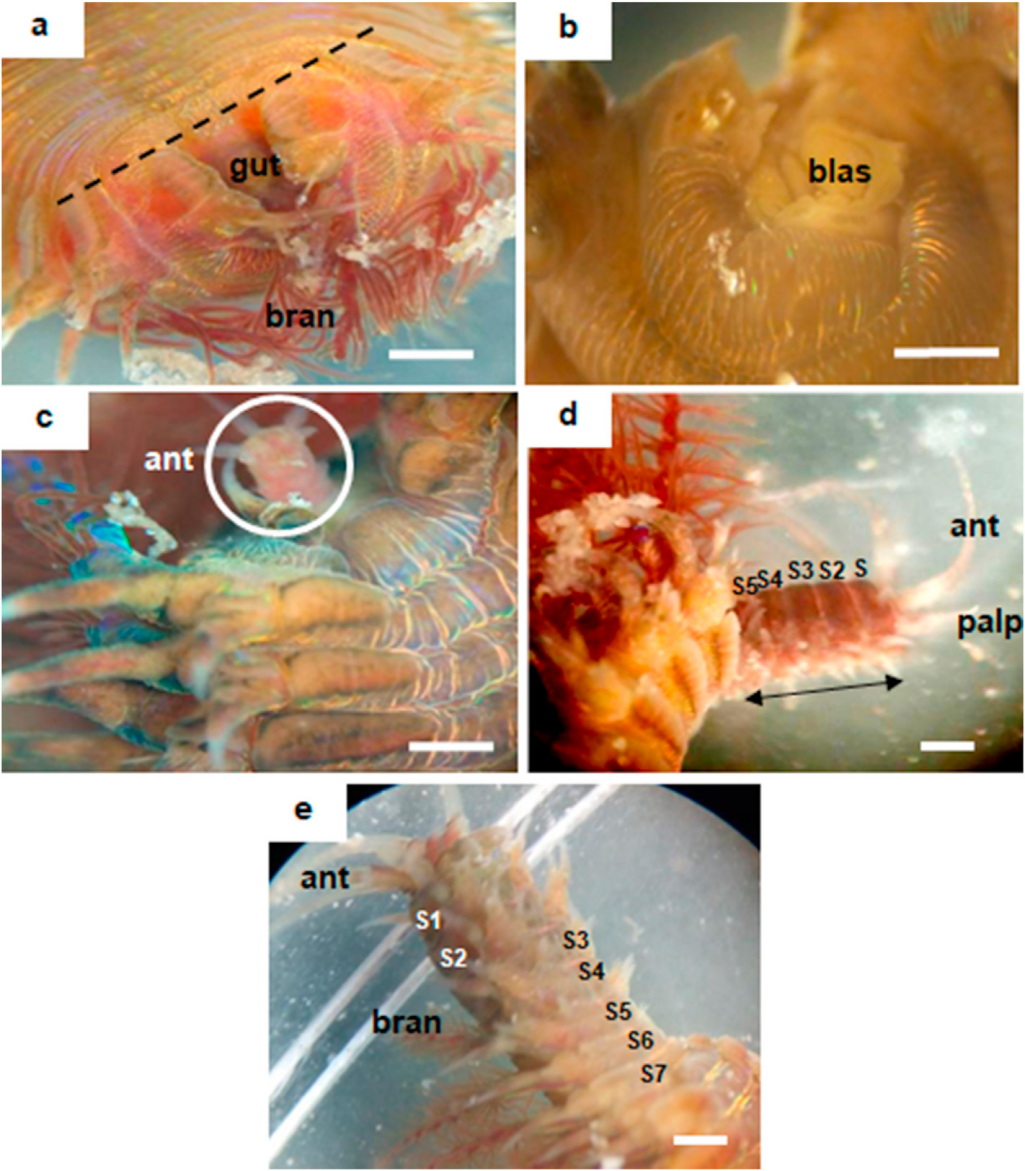
Anterior regeneration in *D. claparedii*. (a) Ventral view. A recovering polychaete at day 1 p.a. Perforated line is where the polychaete was amputated. (b) Axial view. Condition at day 6 p.a. with the presence of blastema tissue (blas) at the mid body plane. (c) Ventral view. Anterior regeneration at day 14 p.a. New head formation with three antennae. (d) Lateral view. Polychaete regenerating at day 30 p.a. Segmentation is obvious as marked by the double-headed arrow. Five new segments arose from four losing segments. (e) Ventral view. Almost complete regeneration at day 60 p.a. Branchiae (bran) was smaller compared to the old body. The mouth has developed and the feeding behaviour has become normal. New segments arose more than the ones amputated. Scale bars = 1 mm.

Prior to the cellular investigation of early NSR, we observed the full anterior regeneration after the polychaete was amputated at the 4^th^ segment (Figure 2). On day 1 post-amputation (p.a.), the amputated site was closed without infection (Figure 3a). We also noticed the appearance of tissue mass at the middle body plane of the amputation site called blastema at day 6 p.a. (Figure 3b). Later on day 14 p.a., we observed new anterior (prostomium and peristomium) appeared, somewhat translucent. The new anterior arose in the middle of the amputation site where the gut is located (Figure 3c). On day 30 p.a., segmentation is seen replacing the lost segments. However, our observation found that new segments were not replacing the exact number of lost segments (Figure 3d). The full anterior regeneration was observed at around day 60 p.a. by comparing the new anterior width with the original body. The widths of the new anterior and the original body were almost the same. However, in the new anterior, branchiae were smaller, and the overall colour of the new anterior was lighter than the original body.

**Figure 4:**
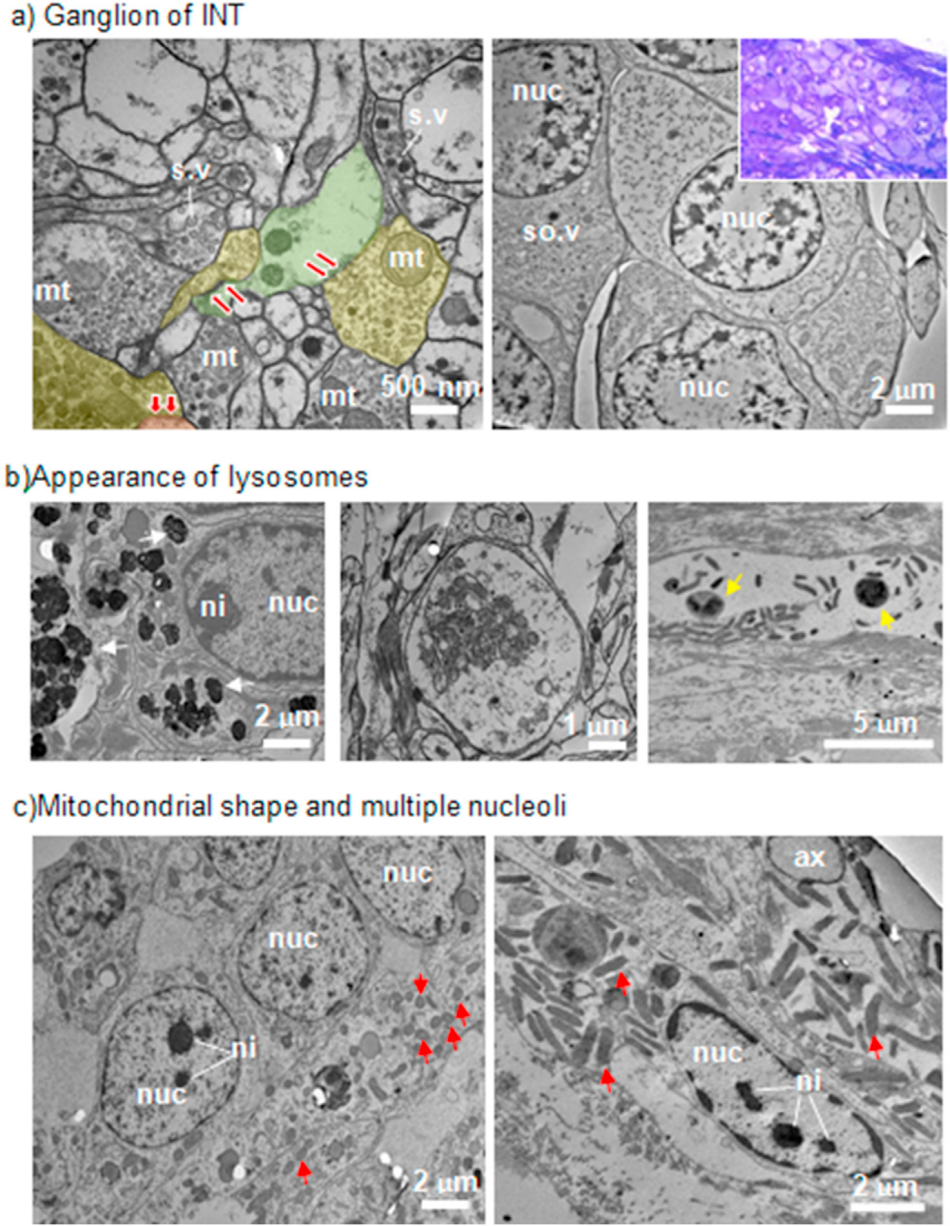
The occurrence of NSR analysed by TEM. (a) Ultrastructures in INT ganglion. Left image: Neuropil showing synaptic transmission between axon terminals (yellow areas) and dendrites (green areas). The transmissions were seen at the synaptic clefts (red arrows). Synaptic vesicles (s.v) are located inside the axon terminals. Right image: Accumulation of perikarya (also from semithin section seen in insert). The nucleus (nuc) is always seen as a prominent structure in a neuron. Perikaryon also performs synaptic connectivity with axon terminal since somatic vesicles (*so.v*) containing neurotransmitter also present there. (b) The appearance of lysosomes in D6 and D14. Left image: Accumulation of lysosomes (white arrows) in blastema cell of D6 polychaete. Middle image: Final stage of lysosomal activity seen in ganglion of D6 polychaete. Right image: Lipofuscins (yellow arrows) in the ganglion of D14 polychaete. (c) Differences in the shape of mitochondria (red arrows) between D6 and D14 polychaetes. Note as well both polychaetes have multiple nucleoli (ni). Left image: Blastema cells (in D6) are recognised by having numerous mitochondria indicating high synthetic activity in the cells. The shape of mitochondria were rounded. Right image: Mitochondria in D14 were elongated in shape and numerous.

The ultrastructure analyses on NSR were conducted on tissues of three groups; ganglion in the intact polychaete (INT), ganglion and blastema in polychaete at day 6 p.a (D6) and ganglion in polychaete of day 14 p.a. (D14). Electron micrograph (Figure 4a) clearly shows the synaptic transmissions between axon terminals and dendrites in the INT ganglion, specifically the neuropil, indicating the occurrence of a neurophysiology process. Furthermore, we also confirmed the presence of perikarya or ganglionic cells from the intact ganglion (Figure 4a). Our TEM findings also revealed distinctive differences between intact and regenerating polychaetes. Compared to INT polychaete, the presence of lysosomes and lipofuscins in both D6 and D14 polychaetes were very apparent, indicating that the cell digestions process was underway (Figure 4b). Interestingly, the presence of numerous lysosomes, as well as secretory granules, were visible in the blastema cells of D6 polychaete. Another distinct feature that we have identified was the presence of numerous mitochondria with various shapes. Mitochondria in ganglia of INT and D6 polychaetes were round in shape, as well as in the blastema. Meanwhile, ganglion in D14 polychaete possessed more elongated mitochondria (Figure 4c). Moreover, multiple nucleoli in the blastema cells of D6 and perikarya of D14 polychaetes are also observed (Figure 4c).

### 3.3 Molecular evidence of early NSR in *D. claparedii*

**Figure 5:**
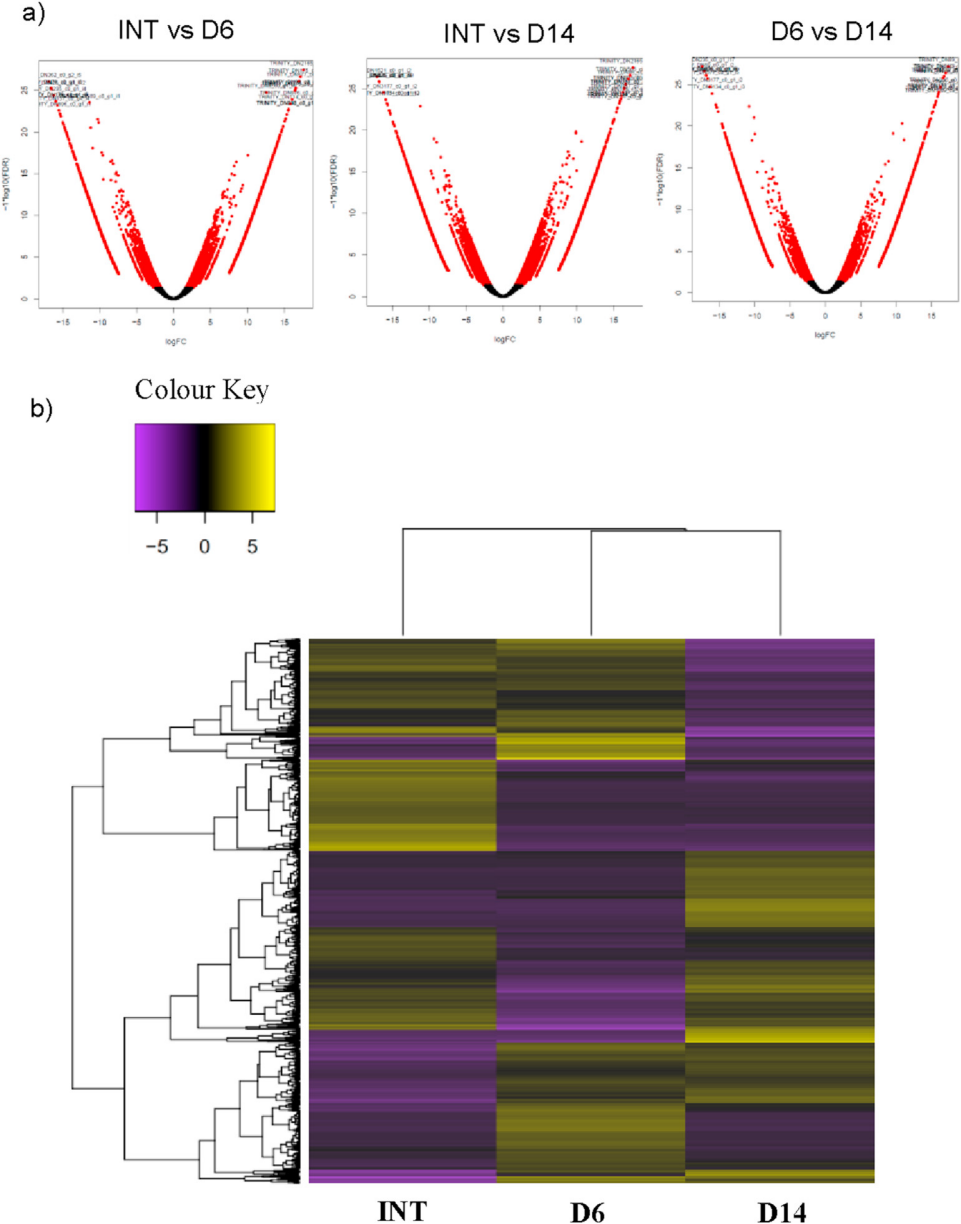
Differential expression (DE) maps for all RNA-seq samples. The analysis was conducted by edgeR Bioconductor package provided by using Trinity. (a) DE between all groups by volcano plots. Red dots show the differences of transcripts between groups are statistically significant and *vice versa* for black dots (*p* < 0.05). Transcripts in negative values of log fold change (logFC) are downregulated and values of positive indicate the transcripts are upregulated. (b) Another expression of DE by a heatmap (*p* > 1 × 10^−10^). Yellow indicates upregulation and purple for downregulation. n = 2.

We investigated the NSR in our polychaete species by conducting RNA-seq on Illumina sequencer. The tissues from the three regenerating stages, i.e. INT, D6 and D14 (n = 2 each group stage) were extracted for their mRNAs prior to their conversion to six libraries. Phix-174 was used as quality and calibration control for Illumina sequencing. Each sequence generated from Illumina sequencing platform was screened against phix-174 to remove the contaminants. RNA-seq analysis has identified 147,445 differential expressed genes (transcripts) with *p* < 0.05. The average contig length was 890.58 bp. Figure 5a shows differentially expressed transcripts (red dots) when compared between groups with *p* < 0.05. Moreover, Figure 5b shows differentially expressed transcripts between groups with *p* < 1 × 10-10, indicating of up- and down-regulation. The transcripts were filtered using CDHit, and 116,203 non-redundant transcripts were obtained. These transcripts were then predicted for protein sequences by Transdecoder, and a total of 37,248 coding sequences were recorded.

### 3.4 Differential expression of proteins related to NSR

The analysis conducted towards transcriptome in the regenerating anterior of *D. claparedii* has identified multiple proteins. Gene Ontology (GO) mapping was conducted to retrieve candidate GO terms associated with the hits obtained after a BLAST search. Then, the proteins were mapped by using Blast2GO (BioBam, Valencia, Spain) to obtain the functional labels associated with the processes that occur in the extracted tissues. Specifically for NSR, GO terms (related to biological function) were listed as i) neurogenesis, ii) nervous system development, iii) central nervous system development, iv) neuron migration, v) regulation of neurogenesis, vi) axon regeneration, vii) positive regulation of axon regeneration, viii) axon extension, ix) regulation of axon extension, x) neuron projection regeneration, xi) axonogenesis, xii) neuron development, xiii) axon development, xiv) generation of neurons, xv) neuron differentiation, xvi) peripheral nervous system development. The sequences obtained have been submitted to NCBI (Table 1).

**Table 1:** Sequencing data accession numbers (https://dataview.ncbi.nlm.nih.gov/object/PRJNA663357?reviewer=i8oa2u3reioo342o8v9gnf5ahc).

No.	Accession No.	Title	BioProject	BioSample	Library ID
1	SRR12672111	Intact segment 4 to 5 of anterior body: replicate 1	PRJNA663357	SAMN16131181	INT-1
2	SRR12672110	Intact segment 4 to 5 of anterior body: replicate 2	PRJNA663357	SAMN16131182	INT-2
3	SRR12672109	Day 6 post amputation replicate 1	PRJNA663357	SAMN16131183	D6-1
4	SRR12672108	Day 6 post amputation replicate 2	PRJNA663357	SAMN16131184	D6-2
5	SRR12672107	Day 14 post amputation replicate 1	PRJNA663357	SAMN16131185	D14-1
6	SRR12672106	Day 14 post amputation replicate 2	PRJNA663357	SAMN16131186	D14-2

**Figure 6:**
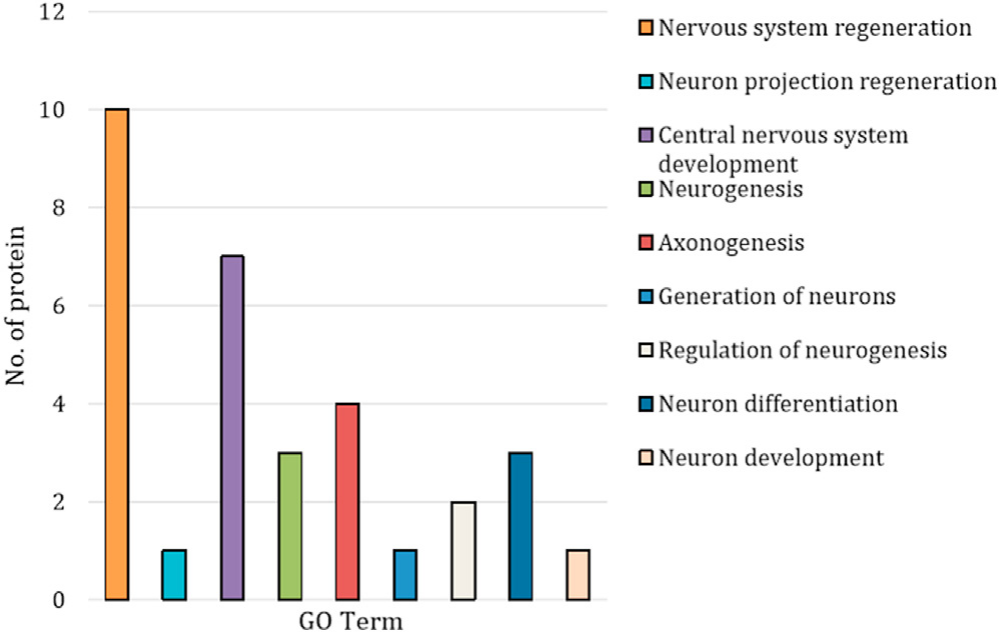
Number of proteins in each GO term. Among nine categories, nervous system development (GO:0007399) has the highest number of proteins up- and downregulated. Noelin-like isoform X2 and noelin-like isoform X3 were among the highly expressed and upregulated during NSR.

Table 2 summarises the proteins expressed during nervous system regeneration based on the GO terms listed, which are significantly different (p < 0.05) compared between groups and exist at -2 > logFC >2. The highest protein expressed and upregulated during NSR in *D. claparedii* was noelin-like isoform X2, which categorised under nervous system development (GO:0007399). The protein was differentially expressed in both D6 and D14 polychaetes compared to INT. In contrast, smoothened homolog was highly downregulated in both D6 and D14 compared to INT based on its logFC. This protein was categorised under GO:0031102, i.e. neuron projection regeneration. Also, several proteins were upregulated and downregulated concurrently. However, the expression occurred in different regenerating polychaetes. For instance, proteoglycan 4 was upregulated in D6 polychaete when compared to INT polychaete. In contrast, the same protein was downregulated in D14 when compared to D6. The phenomenon is interesting because proteoglycan 4 only upregulated at the initiation of regeneration, then downregulated about a week after. Amongst all proteins that satisfied the significant level and logFC, the highest number of protein is for the nervous system development. A total of five proteins identified for upregulated and another five proteins were identified for downregulated processes (Figure 6).

**Table 2:** Selected Genes Ontology categories represented along the anterior NSR. All differences significant compared to INT, except ∗ significant when compared with D6.

GO Term	Description	Protein Name	Group (n = 2) (*P* value)	
			Up-regulation	Down-regulation
GO:0007399	nervous system development	noelin-like isoform X2	D6 (2.72E-23) D14 (2.12E-23)	
		polycomb complex protein BMI-1-B-like isoform X2		D6 (6.23E-14) D14 (1.54E-07)
		polycomb complex protein BMI-1		D6 (9.21E-13) D14 (1.29E-08)
		trafficking protein particle complex subunit 6B		D6 (1.34E-09) D14 (6.69E-08)
		noelin-like isoform X3	D14 (8.84E-07) ∗D14 (0.0002779)	
		proteoglycan 4	D6 (1.83E-08)	∗D14 (1.72E-06)
		protein unc-119 homolog B-like	D6 (9.48E-06)	∗D14 (1.45E-05)
		protocadherin alpha-11-like		D14 (0.0196149)
		zinc finger protein 335 isoform X1		D6 (0.0010263)
		glycoprotein 3-alpha-L-fucosyltransferase A	D14 (0.0001255)	
GO: 0031102	neuron projection regeneration	smoothened homolog		D14 (1.66E-12) D6 (9.62E-09)
GO:0007417	central nervous system development	low-density lipoprotein receptor-related protein 4-like	D14 (1.83E-11)	
		hyaluronan and proteoglycan link protein 3-like		D6 (9.30E-05) D14 (0.0230912)
		versican core protein-like	D6 (2.05E-06)	
		hyaluronan and proteoglycan link protein 1-like	D14 (8.19E-05)	
		achaete-scute homolog 1		D14 (0.0124250)
		versican core protein	D14 (0.0004928)	
		transcription factor SOX-8		D14 (0.0001256)
GO:0022008	neurogenesis	neuron navigator 2 isoform X9	D14 (0.0005557) ∗D14 (0.0059102)	∗D14 (6.40E-09) D14 (0.0004403)
		BTB/POZ domain-containing protein 6-like	D14 (1.12E-07)	
		transcription factor SOX-8		∗D14 (7.45E-05) D14 (0.0001256)
GO: 0007409	axonogenesis	cartilage matrix protein		D14 (2.75E-08) ∗D14 (1.19E-05)
		slit homolog 1 protein	D6 (4.00E-07) D14 (3.45E-07)	
		immunoglobulin A1 protease	D14 (8.70E-05) D6 (0.0085116)	
		cartilage matrix protein	D14 (0.0043983) ∗D14 (0.000296)	
GO: 0048699	generation of neurons	protein IMPACT isoform X1	D14 (4.28E-06)	
GO:0050767	regulation of neurogenesis	EGF-like domain-containing protein 1	D14 (3.47E-07) ∗D14 (3.23E-05)	
		microfibril-associated glycoprotein 4-like isoform X1		D6 (5.43E-07)
GO: 0030182	neuron differentiation	receptor-type tyrosine-protein phosphatase delta-like	D6 (0.0001185)	
		protein Wnt-16-like	∗D14 (0.0014780)	D6 (2.23E-07)
		protein Wnt-5b-like		D14 (7.45E-05) ∗D14 (4.53E-05)
GO: 0048666	neuron development	achaete-scute homolog 1		D14 (0.0039118) D14 (0.0124250)

## 4 Discussion

Here we have investigated the two essential aspects of understanding NSR in *D. claparedii*. The first part involved the characterisation of ANS in intact polychaete and second, aspect regarding cellular and molecular of NSR after the worm lost its anterior section entirely, including the brain.

We have confirmed that *D. claparedii* has a complex nervous system by having both CNS built by a brain and a nerve cord and PNS that constructing the lateral nerves. Our study provides a comprehensive description of the nervous system in this species, representing Onuphidae family, which belongs to order Eunicea. The closest research was done in 1995, covering other species within Eunicea, but only focused on the cephalic nervous system (Orrhage, 1995). The brain observed in *D. claparedii* was very obvious with symmetry shape and vivid appearance of perikarya surrounding the neuropil. Some scientists referred to different names for the brain rather than the ‘brain’ itself. For instance, because of the accumulation and arrangement of the cell bodies/perikarya, terming either supraesophageal ganglion or cerebral ganglion (Müller et al., 2003; Brinkmann and Wanninger, 2009). Interestingly, in *D. claparedii*, there were branches of nerves innervating out of the brain, as opposed to the human brain.

Also, palp nerves, which function for sensing (Purschke, 2005) were found at the anterior of the brain and circumpharyngeal connectives, creating a loop-like nerve connecting down to the nerve cord. The nuchal organs, a pair of ciliated spots for chemosensory and present in all polychaetes, were also observed (Purschke, 2005). The triple-stranded nerve cord in *D. claparedii* is also present in other polychaete species (Helm et al., 2018) We also noticed that lateral nerves were originated from the segmental ganglion of each segment. The three-dimensional analysis revealed the main nerve structures' location, i.e., the brain and the ganglionated nerve cord. The brain was located dorsally at the tip of prostomium above the mouth, while the nerve cord was found ventrally connected to every segment. However, the use of micro-CT does not allow us to distinguish other nerve branches as seen in the conventional histology, including the combination of immunohistochemistry and confocal laser scanning microscope (IHC + CLSM). The inability of micro-CT to distinguish nerve branches were also reported in several studies (Faulwetter et al., 2013; Parapar et al., 2017). Images of soft tissue such as nerves are usually are not well recorded due to low intrinsic X-ray contrast on non-mineralised tissue (Thompson et al., 2020).

Through TEM, perikaryon was observed to contain the prominent nucleus, numerous mitochondria, and synaptic vesicles. The presence of neuropil indicated a complex connection of synapses between axon terminals and dendrites, enabling synaptic transmissions to occur. The vesicles of neurotransmitters or neuropeptide were also observed, suggesting the effect of vesicular volume on exocytosis (Sombers et al., 2004). The observation is similar to the previous study on annelid regeneration from a cellular perspective (Bely, 2014). However, previous study was limited up to blastema formation, while in the present study, we extended this to the early segmentation stage, which was seen in D14 polychaete. For a regeneration process to occur, blastema should start to redifferentiate into specific structures (Bely, 2014). Our findings from TEM magnified the ultrastructures that appeared in blastema tissue. The apparent appearance of lysosomes was seen by electron-dense materials. Lysosome consists of degradative enzymes that break down unwanted biomolecules during cellular metabolism, including regeneration. Furthermore, lysosomes also store Ca^2+^, which can be released into the cytoplasm for intracellular functions (Hesketh et al., 2018). Numerous mitochondria were also apparent in both D6 and D14 polychaetes; however, their shapes changed along the time. Mitochondria in D6 polychaete was round, while the ones in D14 were elongated. A round-shaped mitochondrion can produce more reactive oxygen species (Ahmad et al., 2013), which involves in apoptosis during the wound healing process (Bryan et al., 2012; Cano Sanchez et al., 2018). The presence of elongated mitochondria indicate protection from degradation. The mitochondria have more cristae, meaning increased ATP synthase activity, thus maintaining the cells' viability to continue regenerating. This condition is known as a cellular stress response (Fulda et al., 2010). Another indication that cells were actively regenerating was the appearance of multiple nucleoli in the nucleus (Pedersen, 1972; Maki et al., 2007). Nucleolus composed of filamentous and granular structures and production site of ribosomal RNA. The RNA then will produce ribosomal protein to become ribosomal subunit (Eeken, 2001), which is crucial in producing specific proteins for regeneration.

The transcriptomes of the NSR in amputated *D. claparedii* in two regenerating stages, as well as intact polychaetes, were sequenced and compared to each other. Through this experiment, we have identified proteins involved in NSR and other processes related to the production of new nerve tissues. Our finding has identified nine categories from GO terms that satisfied the significant level (*p* < 0.05) and fold change (≥ 2 & ≤ -2). Under the nervous system development (GO: 0007399), five proteins were upregulated. The highest differentially expressed and highest fold change belonged to the noelin-like isoform X2, a putative protein for noelin (OLFM1). We also found that noelin-like isoform X3 is among the highest upregulated protein that differentially expressed. Noelin or olfactomedin (Anholt, 2014) belongs to olfactomedin domain-containing proteins and is involved in neuronal differentiation during development, neurogenesis, and generation of neural crest during embryogenesis and larval development (Moreno and Bronner-Fraser, 2005; Rice et al., 2012). Olfactomedin 1 was also reported to suppress the cleavage of amyloid precursor protein and inhibit the subsequent production of amyloid-beta, the main contributor to Alzheimer's disease (Takahama et al., 2004). Furthermore, slit homolog 1 protein was also expressed in the regenerating polychaetes. This protein is reportedly involving in axon guidance or navigation during cellular migration (Wong et al., 2002). This protein has not been documented anywhere except in an article by Ribeiro et al. (2019) who studied the anterior regeneration in a syllid worm. Protein unc-119 homolog B-like is similar to unc-119 that functioning in neural architecture patterning. This protein is well conserved in metazoa, and the mutation to this protein will lead to nervous system disorientation and paralysis (Manning et al., 2004). However, this protein was downregulated in D14 polychaete that might show neural architecture patterning has been completed.The last relevant protein in NSR is neuron navigator 2 isoform X9 which might play a role in cell migration and axonal outgrowth (Maes et al., 2002; Muley et al., 2008) and it was significantly expressed in D14 which probably true since the nervous system was in the process of development.

## 5 Conclusions

In summary, we have presented the characteristics of the anterior nervous system in *D. claparedii* morphologically, i.e. the region for anterior NSR. We have highlighted the brain and ventral nerve cord as the main structures in CNS. We further investigated the ultrastructures, particularly the ganglion of the nerve cord from the regenerating and intact tissues and concluded significant differences between the regenerating stages. Our molecular data also revealed new putative proteins responsible in nervous system regenerating requiring further extensive investigation as further experimentation will provide complementary information on questions such as real-time protein activities during nervous system regeneration.

## Declarations

### Author contribution statement

Mohd Ulul Ilmie Ahmad Nazri, Wan Iryani Wan Ismail: Conceived and designed the experiments; Performed the experiments; Analyzed and interpreted the data; Contributed reagents, materials, analysis tools or data; Wrote the paper.

Mohd Hafizi Mahmud, Basri Saidi, Mohd Noor Mat Isa, Zolkapli Ehsak: Performed the experiments; Analyzed and interpreted the data; Contributed reagents, materials, analysis tools or data; Wrote the paper.

Othman Ross: Conceived and designed the experiments; Wrote the paper.

Izwandy Idris: Conceived and designed the experiments; Analyzed and interpreted the data; Contributed reagents, materials, analysis tools or data; Wrote the paper.

### Funding statement

This work was supported by the 10.13039/501100003093Ministry of Higher Education (MOHE), FRGS/1/2016/WAB09/UMT/02/2.

### Data availability statement

Data associated with this study has been deposited at NIH under the url https://dataview.ncbi.nlm.nih.gov/object/PRJNA663357?reviewer=i8oa2u3reioo342o8v9gnf5ahc.

### Declaration of interests statement

The authors declare no conflict of interest.

### Additional information

No additional information is available for this paper.
